# Energy Drink Consumption Among Adolescents in Northern Spain: Habits, Risk Perception and Associated Factors

**DOI:** 10.3390/nu18081240

**Published:** 2026-04-15

**Authors:** Maria del Mar Fernandez-Alvarez, Judit Cachero-Rodríguez, Cristina Fernández-Rodríguez, Carla Carrizo-Rodríguez, María García-Martínez, Ruben Martin-Payo

**Affiliations:** 1 Faculty of Medicine and Health Sciences, University of Oviedo, 33006 Oviedo, Spain; fernandezmar@uniovi.es (M.d.M.F.-A.); uo291107@uniovi.es (C.F.-R.); uo292403@uniovi.es (C.C.-R.); martinruben@uniovi.es (R.M.-P.); 2 Instituto de Investigación Sanitaria del Principado de Asturias (ISPA), 33006 Oviedo, Spain; 3 Faculty of Nursing, University of Santiago de Compostela, 15782 Santiago de Compostela, Spain; maria.garcia.martinez@usc.es; 4 CLINURSID Research Group, University of Santiago de Compostela, 15705 Santiago de Compostela, Spain

**Keywords:** energy drinks, adolescents, consumption patterns, risk perception, health behaviors, Spain

## Abstract

**Background/Objectives**: Energy drinks (EDs) are widely consumed by adolescents despite increasing evidence of adverse health effects. This study aimed to assess ED consumption patterns, risk perception, and associated factors among adolescents in the Principality of Asturias, Spain. **Methods**: A cross-sectional descriptive study was conducted between January and March 2025 in a sample of 1250 adolescents aged 13–18 years. Data were collected using an anonymous questionnaire assessing frequency and age at initiation of ED consumption, motives and contexts of use, perception of adverse effects, and co-occurrence with other risk behaviors. Descriptive analyses and linear regression models were performed to identify predictors of ED consumption. **Results**: Overall, 29.6% of participants reported occasional or habitual ED consumption. Consumption was significantly higher among upper secondary school students, particularly in social settings and during study-related activities (*p* < 0.001). ED consumption was significantly associated with other risk behaviors, including alcohol and tobacco use (*p* < 0.001). Additionally, 8.6% of adolescents reported that EDs have no adverse health effects. Male sex, alcohol consumption, and tobacco use were the main predictors of ED consumption. **Conclusions**: A substantial proportion of adolescents consume EDs, with early initiation and increasing consumption with age and educational level. Although some adverse effects are recognized, risk perception remains low. These findings underscore the need for preventive, educational, and regulatory strategies to reduce ED consumption and its normalization among adolescents.

## 1. Introduction

Energy drinks (EDs) are defined as nonalcoholic beverages characterized by their content of caffeine, sugars, vitamins, taurine, and other compounds with stimulant effects [1,2]. In recent years, their consumption has increased exponentially, particularly among adolescents and young adults [3,4,5]. Globally, it is estimated that more than half of the population has consumed EDs at least once [4]. In Europe, the prevalence of ED consumption among adolescents reaches 68% [2], and in Spain, data from the Survey of Drug Use in Secondary Education (ESTUDES) indicate that 47.7% of students aged 14–18 years consumed EDs in the previous 30 days, a proportion similar to that observed in the Principality of Asturias (46.4%) [6].

Although EDs are marketed for their purported benefits in enhancing physical and mental performance, reducing fatigue, and improving concentration, these claims contrast with growing scientific evidence of adverse health effects. The literature demonstrates a strong dose–response relationship between the amount consumed and the occurrence of harmful outcomes [7]. ED consumption has been associated with cardiovascular disturbances [1,8,9], gastrointestinal symptoms [8,10], anxiety [1], stress and depressive symptoms [10,11,12,13,14], sleep problems [1,8,10,11], and increased insulin resistance [11], which may contribute to the development of type 2 diabetes mellitus. In addition, negative effects on oral health have been reported, including enamel erosion and dental caries [15], as well as associations with unhealthy dietary patterns, such as frequent fast-food consumption [14].

These consequences are particularly concerning during adolescence, a developmental period characterized by increased emotional vulnerability, a strong need for social acceptance, and heightened susceptibility to external influences. In this context, several studies have shown a synergistic relationship between ED consumption and other risk behaviors, including tobacco use [5,11,14,16], use of other drugs [16], and mixing EDs with alcohol [2,11,14,16,17,18,19]. This practice is often intended to counteract the sedative effects of alcohol through the stimulant action of caffeine [7,10]. Furthermore, ED consumption has been linked to violent behaviors [14,20], self-harm [8], delinquent behaviors [11], and even suicidal ideation [14].

Despite this evidence, adolescents’ perception of the risks associated with ED consumption remains low [1], and these beverages continue to be widely accessible. This low risk perception may be partly explained by marketing strategies that associate ED consumption with athletic success and social enjoyment [21], reinforcing the mistaken belief that these products are harmless or even healthy [22]. Consistent with this, previous studies have identified the main reasons for ED consumption as the desire to increase energy, stay alert, improve performance, enjoyment of taste, and use in social interactions [2,8,19,23,24,25].

In response to this situation, several countries have implemented legislative measures aimed at limiting ED consumption among minors, including sales restrictions, specific taxation, warning labels, and advertising limitations [18,26,27]. In Europe, Regulation (EU) No. 1169/2011 [28] requires warning labels indicating high caffeine content and specifying the amount per 100 mL. However, despite these regulations, ED brands have adapted their marketing strategies to the digital environment, using social media, sports sponsorships, and influencer collaborations to increase their reach among younger audiences [29]. Advertising on social media is particularly relevant, as these platforms are sometimes adolescents’ primary source of information about EDs [30]. Moreover, exposure to digital advertising has been associated with increased consumption among children and adolescents [31], independent of screen time [32]. EDs are among the most frequently mentioned products in digital environments [33], and in most cases, content is not subject to age restrictions [34].

Given the high prevalence of ED consumption among adolescents, the associated health risks, and the influence of social and commercial environments, it is essential to characterize current consumption patterns in specific settings. Therefore, the objective of the present study was to analyze ED consumption among adolescents attending several secondary schools in the Principality of Asturias, with the aim of providing evidence to inform health promotion strategies and encourage healthy behaviors in this population.

## 2. Materials and Methods

### 2.1. Study Design

A descriptive cross-sectional study was conducted among school-attending adolescents in the Principality of Asturias (Spain). The study was carried out between January and March 2025. This manuscript was prepared in accordance with the STROBE checklist for descriptive studies [35].

### 2.2. Participants

The study population consisted of adolescents aged 13–18 years enrolled in public and publicly funded private schools in the Principality of Asturias. In the Spanish educational system, Secondary Education (ESO) includes students aged 13 to 16, while High School (BACH) includes those aged 17 and 18. The inclusion criteria were: (i) provision of written informed consent signed by parents or legal guardians for students younger than 16 years, or by the students themselves if they were aged 16 years or older; and (ii) absence of physical or mental limitations that could prevent completion of the questionnaire.

The study protocol was approved by the Research Ethics Committee of the Principality of Asturias (registration number CEImPA 2024.531, 30 December 2024).

### 2.3. Data Collection

Participant recruitment was conducted through the educational centers where adolescents were enrolled. Members of the research team contacted 9 public and publicly funded private schools, selected by convenience sampling, from both urban and rural areas. The informed consent forms and information sheets were sent to the schools and distributed by teaching staff to legal guardians or students, depending on participants’ age.

Once informed consent was obtained, data collection was conducted in person using an anonymous questionnaire. Depending on each school’s preference, the questionnaire was administered either in paper format or digitally through an online form using Microsoft Forms.

Based on the questionnaire used in the study “Gathering consumption data on specific consumer groups of EDs” conducted by the European Food Safety Authority (EFSA) [2], the ESTUDES survey [6], and the available literature [36], an ad hoc questionnaire was developed. A semantic validation (wording and comprehension) and content validation (clarity and relevance) were carried out. The questionnaire included personal characteristics (age, sex, and school grade), as well as items related to the frequency and patterns of tobacco consumption (never, occasionally–non-regular, limited to specific occasions–, weekends, or daily), alcohol consumption (never, weekends, 2–3 non-weekend days per week, ≥4 non-weekend days per week), screen use (never, <1 h per day, 1–3 h per day, 3–5 h per day, >5 h per day), sleep duration (<7 h/per night; 8 or more hours per night) and ED consumption (never, occasionally–non-regular, limited to specific occasions–, habitually/weekly, or daily). The questionnaire also included items on engagement in other risk behaviors and the perception of adverse effects associated with consumption (no adverse effects, increased heart rate, tremors, gastrointestinal disorders, seizures, increased blood pressure, anxiety, irritability, depression, hyperactivity).

Regarding consumption habits, students were asked about their reasons for consuming EDs (need energy, stay awake, like the taste, improve concentration, enhance performance or functional motivations such as increasing energy, staying awake, or improving alertness, rather than exclusively cognitive or academic enhancement, it is trendy, other), the contexts in which they consumed them (at home, with friends, in bars, in nightclubs, during sports activities, while studying, other), and whether they consumed EDs mixed with alcohol or tobacco (yes/no). Questions related to the age at initiation of ED consumption were also included.

### 2.4. Statistical Analysis

Descriptive analyses of sociodemographic and personal variables, as well as variables related to the study objectives, were performed and expressed as frequencies, percentages, means, and standard deviations (SD). The Kolmogorov–Smirnov test was used to assess normality of data distribution. Differences between school grade, ESO or BACH, were analyzed using the chi-square test. Linear regression analysis was conducted to identify predictors of ED consumption among adolescents, adjusted for age, sex, educational level, alcohol consumption, and tobacco use.

The level of statistical significance was set at *p* ≤ 0.05. All analyses were performed using IBM SPSS Statistics software (Armonk, New York, NY, USA), version 27.0.

## 3. Results

### 3.1. Sample Characteristics

A total of 1250 students from 9 educational centers (public and publicly funded private schools) in the Principality of Asturias participated in the study. The mean age of participants was 15.13 years (SD = 1.38). The mean age of students in Secondary Education (ESO) was 14.32 years (SD = 0.99), whereas the mean age of High School students (BACH) was 16.55 years (SD = 0.64). The remaining characteristics of the sample are shown in Table 1.

A total of 29.6% of adolescents (N = 369) reported occasional or regular consumption of EDs. Participants were asked about the age at which they first tried EDs. Overall, 7% (N = 54) reported first consumption between 6 and 9 years of age, 38.1% (N = 293) between 10 and 12 years, 43.2% (N = 332) between 13 and 14 years, and 11.7% (N = 90) at 15 years of age or older.

### 3.2. Consumption Habits Related to EDs

No statistically significant differences were observed in the reasons for ED consumption according to educational level. However, consumption in specific settings was significantly higher among high school students compared to secondary education students, specifically in bars (12.7% ESO vs. 27.8% BACH), nightclubs (8.8% ESO vs. 31.3% BACH), and during studying (6.4% ESO vs. 14.6% BACH) (Table 2).

The analysis of concurrent ED consumption and other risk behaviors showed significant differences between the two educational levels across all evaluated variables (*p* < 0.001) (Table 3).

### 3.3. Perceived Risk Associated with ED Consumption

The majority of participants reported increased heart rate (91.9%), hyperactivity (83.2%), and high blood pressure (62.2%) as perceived risks of ED consumption (Table 4). Unexpectedly, 8.6% reported no perceived risk of ED consumption.

### 3.4. Factors Associated with ED Consumption

Regression analysis showed that ED consumption was 3.1 times more likely when consumed with alcohol and 1.9 times more likely when consumed with tobacco, respectively. Males were twice as likely as females to consume EDs (Table 5).

## 4. Discussion

The results of the present study show that overall ED consumption—considering both occasional and habitual use—among adolescents in the Principality of Asturias is lower than that reported in Europe [2] and in previous studies conducted in Spain [6,36]. However, when habitual consumption alone is analyzed, the findings are comparable to those reported in earlier research [36,37,38]. Trapp et al. [37] observed a weekly consumption rate of 9.5% among Australian adolescents, whereas Schröder et al. [38] and Cruz-Muñoz et al. [36] reported prevalences of 12.3% and 13.5%, respectively, among Spanish adolescents.

The differences observed in overall ED consumption prevalence compared with international organizations and previous national studies may be explained by cultural factors, as the popularization of these beverages in the study area may have occurred more recently than in other European regions, or by methodological variations across studies.

In contrast, habitual consumption appears to be similar to that reported in recent investigations, suggesting that this pattern is more stable internationally and that frequent consumers maintain similar behaviors regardless of local context. This finding reinforces the consideration of habitual ED consumption as a public health concern requiring intervention. Future studies should focus on identifying the determinants of ED consumption in order to design targeted preventive strategies.

Regarding age at initiation, 38.1% of adolescents reported first trying EDs between 10 and 12 years of age, consistent with findings from other studies that place the mean age of initiation at approximately 10 years [37]. At both regional and national levels, an increase in ED consumption among younger adolescents has been observed [5,6], along with an increasingly early initiation pattern [2]. This finding should be taken into account when designing interventions, as prevention efforts need to address populations younger than the typical age of initiation in order to be effective.

The main reasons for ED consumption reported by adolescents in this study are consistent with previous findings in the literature, including preference for taste, the need for energy, and the desire to stay awake [2,7,8,11,23,24,36,37,39]. In some cases, preference for these beverages may become an acquired behavior through repeated exposure, a phenomenon known as the mere exposure effect, whereby repeated consumption of a palatable product increases the likelihood of continued use [40]. This repetition effect may contribute to the consolidation of consumption habits. Additionally, the need for energy and the desire to stay awake reflect a primarily functional pattern of use related to academic or social demands, reinforcing the perception of EDs as cognitive performance–enhancing tools. This interpretation is consistent with the findings of Kaldenbach et al. [39], who identified a strong association between ED consumption and the pursuit of wakefulness during periods of intensive study. The need for concentration and performance enhancement has also been identified as a relevant motivation in previous studies, suggesting that academic pressures play an important role in ED consumption [8,19,24,37].

The results also show differences in the timing and settings of ED consumption according to educational level, suggesting that variations in consumption depend not only on age but also on context. Upper secondary school students reported significantly higher consumption in bars and nightclubs compared with students in compulsory secondary education, consistent with findings reported by Cruz-Muñoz et al. [36]. This pattern is expected, as older adolescents are more likely to participate in nightlife activities. Consumption with friends was also one of the most frequently reported contexts, highlighting the influence of the social environment as a key determinant, in line with previous studies identifying leisure activities and social interactions as central elements in ED consumption during adolescence [7,8,11,19,23,24,39].

In the present study, a significant association was observed between ED consumption and alcohol use, with higher prevalence among adolescents at a higher educational level. This could be influenced by the legal prohibition in Spain on the sale of alcohol to individuals under 18 years of age. Nevertheless, the pattern observed in the present study reflects trends described in previous research indicating higher EDs and alcohol consumption among older adolescents [19,36]. Similar findings have been reported in both international and national populations, including the study by Doggett et al. [17] and the ESTUDES survey [6], which indicate that 13.2% and 19.5%, respectively, of Spanish adolescents have mixed EDs with alcohol. The available evidence suggests that this behavior is widespread and of concern, as the combination of EDs and alcohol may mask the depressant effects of alcohol and increase the risk of engaging in hazardous behaviors [7,8,14]. In this regard, the systematic review by Marinoni et al. [20] highlights the close relationship between EDs and alcohol consumption among adolescents, while Soós et al. [7] report that adolescents who mix EDs with alcohol are more likely to engage in other substance use, suggesting that this combination may be part of a broader pattern of risk behaviors during adolescence.

An association between ED consumption and tobacco use was also observed, consistent with previous studies such as those by Soós et al. [7] and Ajibo et al. [11], which report a positive relationship between these behaviors in adolescents. Moreover, the simultaneous consumption of EDs, alcohol, and tobacco was significantly correlated, reinforcing the hypothesis that habitual ED consumers tend to engage in a broader pattern of risk behaviors. Previous research suggests that this combination may act as an important predictor of the adoption of other harmful behaviors, including illicit drug use [7,8]. These findings support the need to address ED consumption not in isolation but in conjunction with co-occurring risk behaviors.

In this study, 8.6% of adolescents perceived that EDs have no adverse effects, consistent with findings reported by Sánchez-Sánchez et al. [19], suggesting that limited awareness of risks may influence consumption decisions. This observation aligns with the Health Belief Model, which posits that a lack of perceived risk is associated with reduced engagement in health-promoting behaviors [41]. At the same time, the immediate perceived benefits of EDs, such as increased energy or physical performance, may outweigh risk perception, particularly among adolescents with an athletic profile [22], favoring the perception of EDs as healthy products and promoting their consumption. The literature reports heterogeneous findings in this area. Some studies indicate that EDs are perceived as less healthy among adolescents with higher health awareness [22,42], whereas those with a strong athletic identity and greater exposure to sports-related marketing tend to hold more favorable perceptions of ED consumption [21,22]. Indeed, evidence suggests that exposure to messages emphasizing positive effects on body image through social media has a strong influence on adolescents and promotes consumption of advertised products [43]. In this context, Wierzejska et al. [44] found that more than half of adolescents do not perceive EDs as harmful to their health, and that low risk perception is associated with higher consumption and reduced willingness to modify consumption habits. These findings suggest the need for continued progress in regulating EDs advertising, as motivational factors amenable to intervention appear to exist alongside the need for educational strategies and legislative measures [45].

Finally, alcohol consumption, male sex, and tobacco use were significant predictors of ED consumption, making adolescents approximately 3.1, 2.0, and 1.9 times more likely to consume EDs, respectively, consistent with previous findings and supporting the validity of the present results. Alcohol use emerged as a strong predictor of ED consumption, in line with prior studies [14,16]. This association is particularly concerning, as Doggett et al. [17] reported that adolescents who combine EDs with alcohol have a 3.38-fold higher risk of maintaining this behavior over time. Male sex was also significantly associated with higher ED consumption, consistent with multiple studies [11,38], with some reporting a nearly threefold higher likelihood of ED consumption among males compared with females [19]. Lastly, tobacco use was also confirmed as a predictor, in accordance with the literature indicating that ED consumption during adolescence often co-occurs with smoking [11,13,16,19,38] or vaping [14].

One of the main strengths of this study is the large sample of participating adolescents, which is representative of the adolescent population of this autonomous community, thereby increasing the external validity of the findings. This information is essential for the design and implementation of effective health promotion strategies and interventions aimed at preventing associated risks, promoting healthy lifestyle behaviors, and protecting vulnerable groups such as adolescents. Preventive strategies should ideally involve not only adolescents themselves but also parents, educators, and health professionals. Through a comprehensive and collaborative approach, the long-term adverse effects of this behavior could be mitigated and healthy habits promoted among young people.

This study has several limitations. First, participants were required to select their responses from predefined lists of perceived risks and consumption locations, which may have restricted the range and nuance of their answers. Additionally, open-ended responses had to be categorized by the research team, introducing potential subjectivity into the coding process. The survey instrument had not been previously validated or pre-tested, which may affect the reliability of some measures. Furthermore, schools were allowed to choose between paper-based and online administration formats; this inconsistency in survey delivery could have introduced systematic bias. The study also did not measure the exact amount of caffeine consumed, which can vary substantially across brands, serving sizes, and formulations. Other sources of caffeine intake, such as coffee, tea, soft drinks, or dietary supplements, were not assessed, even though they may influence both consumption patterns and risk perception. Future studies should therefore include precise measurements of total caffeine intake, as well as variables related to body image perception, which may also play a role in adolescents’ motivations and risk appraisal. The cross-sectional design of the study also prevents the establishment of causal relationships between ED consumption and the associated variables. It should also be noted that the higher consumption observed in nightclubs, but not in bars, may be influenced by age-related access restrictions, as individuals under 18 years of age (or 16 in exceptional cases) may be unable to enter certain venues. Finally, recall bias and social desirability bias may have influenced participants’ responses, as adolescents may not accurately remember their ED consumption or may provide answers they perceive as socially acceptable.

## 5. Conclusions

The present study characterizes ED consumption among school-attending adolescents in the Principality of Asturias (Spain), showing that a substantial proportion of adolescents report occasional or habitual consumption, with initiation occurring predominantly at early ages. The results indicate that ED consumption increases with age and educational level. Furthermore, despite recognition of some adverse effects, a proportion of adolescents exhibit a low perception of the risks associated with ED consumption, which may contribute to its normalization. Taken together, these findings underscore the need to address ED consumption from a comprehensive perspective, incorporating early preventive strategies, educational programs aimed at improving risk perception, and regulatory measures to limit their marketing and availability.

## Figures and Tables

**Table 1:** Characteristics of the surveyed adolescents (N = 1250).

Characteristics	Total (N = 1250)	ESO (N = 797)	BACH (N = 453)
Sex % (N)			
Male	49.4 (617)	48.6 (387)	50.8 (230)
Female	50.6 (632)	51.4 (409)	49.2 (223)
School grade % (N)			
2nd year ESO * (13–14 years old)	21 (262)	32.9 (262)	
3rd year ESO * (14–15 years old)	19.3 (241)	30.2 (241)	
4th year ESO * (15–16 years old)	23.5 (294)	36.9 (294)	
1° BACH ** (16–17 years old)	24.5 (306)		67.5 (306)
2° BACH ** (17–18 years old)	11.8 (147)		32.5 (147)
Sleep duration % (N)			
≤7 h	62.3 (773)	56.3 (445)	73 (328)
Screen time % (N)			
Never	1.3 (16)	1.5 (12)	0.9 (4)
<1 h	46.9 (583)	49.1 (389)	43 (194)
1 y 3 h	28 (348)	27.1 (215)	29.5 (133)
3 y 5 h	16.6 (207)	15 (119)	19.5 (88)
>5 h	7.2 (90)	7.3 (58)	7.1 (32)
Tobacco consumption % (N)			
Non-smoker	89.9 (1124)	93.1 (742)	84.3 (382)
Occasionally	6.2 (78)	4.6 (37)	9.1 (41)
Weekends	1.8 (22)	1.4 (11)	3.3 (15)
Daily	2.1 (26)	0.8 (6)	3.3 (15)
Alcohol consumption % (N)			
Never	71.8 (897)	87.2 (695)	44.6 (202)
Weekends	25.9 (324)	10.7 (85)	52.8 (239)
2–3 non-weekend days per week	1.7 (21)	1.4 (11)	2.2 (10)
≥4 non-weekend days per week	6.7 (8)	0.8 (6)	0.4 (2)
ED consumption % (N)			
Never	70.4 (877)	73.1 (581)	65.6 (296)
Ocasionally	17.8 (222)	16.5 (131)	20.2 (91)
Habitually	11.8 (147)	10.4 (83)	14.2 (64)

* Secondary Education (ESO); ** High School (BACH).

**Table 2:** Reasons, locations, and occasions for ED consumption among adolescents (N = 1250).

	Total	ESO *	BACH **	*p*
Reasons for consumption % (N)				
Need energy	41.1 (259)	38.7 (146)	44.7 (113)	0.138
Stay awake	41.4 (264)	38.7 (146)	45.3 (115)	0.101
I like their taste	65.0 (413)	64.4 (246)	66.0 (167)	0.677
Concentration augmenting	15.9 (100)	13.9 (52)	19.0 (48)	0.082
Enhance performance	29.7 (187)	30.0 (113)	29.2 (74)	0.845
It is trendy	11.1 (70)	13.0 (49)	8.4 (21)	0.071
Where they consume them % (N)				
At home	13 (162)	13.4 (106)	12.4 (56)	0.632
Friends	22.3 (278)	21.9 (174)	23.1 (104)	0.633
Bar	18.2 (226)	12.7 (101)	27.8 (125)	<0.001
Nightclubs	17.0 (212)	8.8 (70)	31.3 (142)	<0.001
Sports activities	16.2 (201)	16.4 (130)	15.7 (71)	0.745
While studying	9.4 (117)	6.4 (51)	14.6 (66)	<0.001

* Secondary Education (ESO); ** High School (BACH).

**Table 3:** Proportion of adolescents with concurrent ED consumption and other risk behaviors (N = 1250).

	Total	ESO *	BACH **	*p*
EDs and alcohol % (N)	13.4 (167)	6.8 (54)	25.1 (113)	<0.001
EDs and tobacco % (N)	5.3 (66)	3.8 (30)	8.0 (36)	0.001
Tobacco and alcohol % (N)	8.6 (107)	5.1 (41)	14.6 (66)	<0.001
EDs, tobacco and alcohol % (N)	4.6 (57)	3.0 (24)	7.3 (33)	<0.001
EDs mixed with alcohol in the past month % (N)	14.7 (182)	10.3 (81)	22.5 (101)	<0.001

* Secondary Education (ESO); ** High School (BACH).

**Table 4:** Perceived risks associated with ED consumption (N = 1250).

Adverse Effect	% (N)
Increased heart rate	91.9 (1050)
Hyperactivity	83.2 (950)
Increased blood pressure	62.2 (710)
Anxiety	36.9 (422)
Tremors	34.9 (399)
Gastrointestinal disorders	30.9 (353)
Irritability	20.6 (235)
Seizures	15.2 (174)
Depression	9.3 (106)
No perceived adverse effects	8.6 (108)

**Table 5:** Linear regression analysis predicting ED consumption.

Variable	β	*p*	CI 95%
Alcohol consumption	3.07	<0.001	2.366–3.993
Male sex	2.05	<0.001	1.585–2.653
Tobacco use	1.88	0.003	1.233–2.879

Model adjusted for age, sex, educational level, alcohol consumption, and tobacco use.

## Data Availability

The original contributions presented in this study are included in the article. Further inquiries can be directed to the corresponding author.

## Abbreviations

The following abbreviations are used in this manuscript:

BACH	High School (Bachillerato)
ED	Energy drink
EDs	Energy drinks
EFSA	European Food Safety Authority
ESO	Secondary Education (Educación Secundaria Obligatoria)
ESTUDES	Survey of Drug Use in Secondary Education in Spain (Encuesta sobre Uso de Drogas en Enseñanzas Secundarias en España)
